# Supplementary figures and images for: Wormhole attack detection and mitigation model for Internet of Things and WSN using machine learning
Source: PeerJ Comput Sci. 2024 Aug 28;10:e2257. doi: 10.7717/peerj-cs.2257 (PMC11419650; doi:10.7717/peerj-cs.2257)

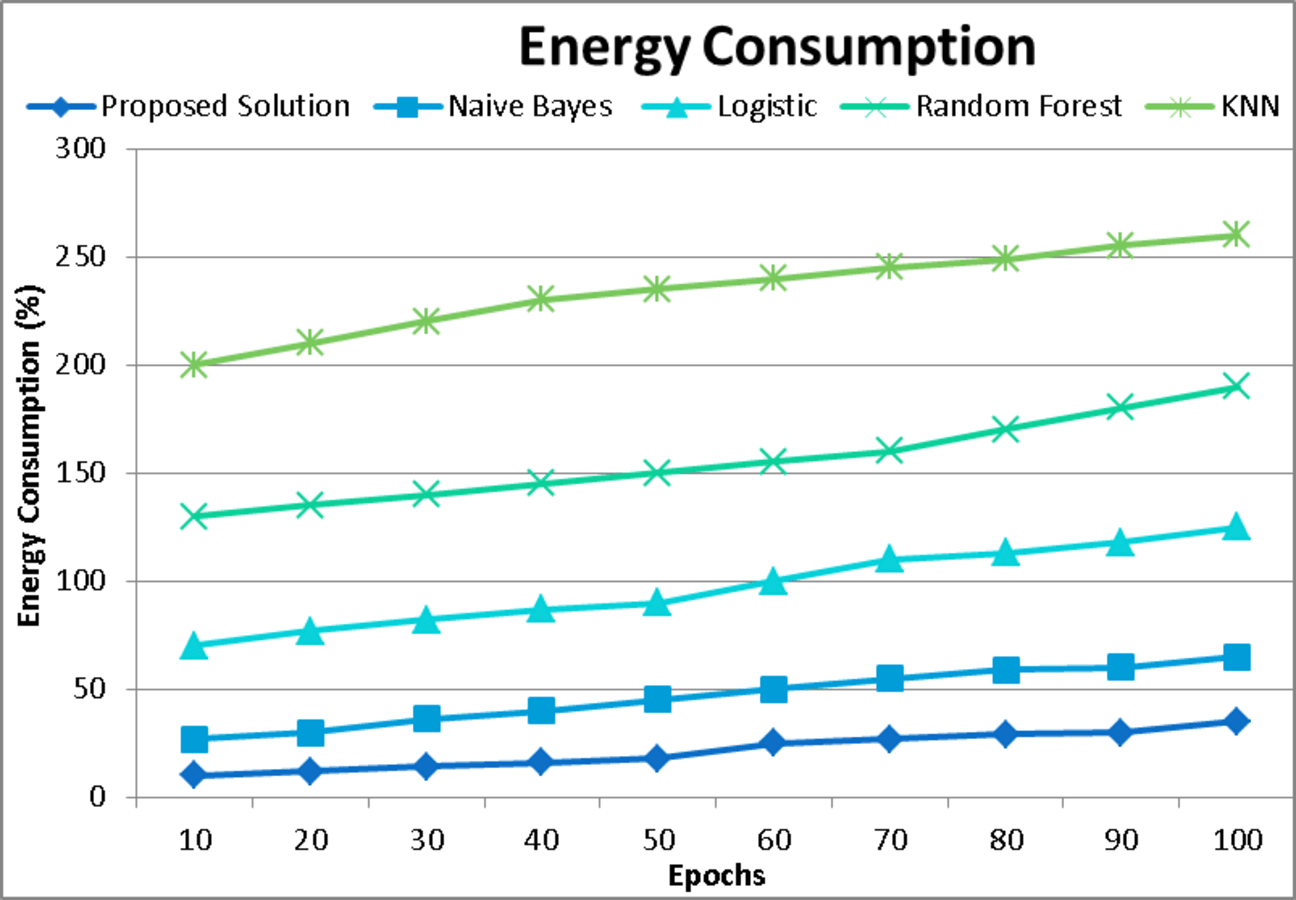

Supplement: Figure S1 [file peerj-cs-10-2257-s002.png]

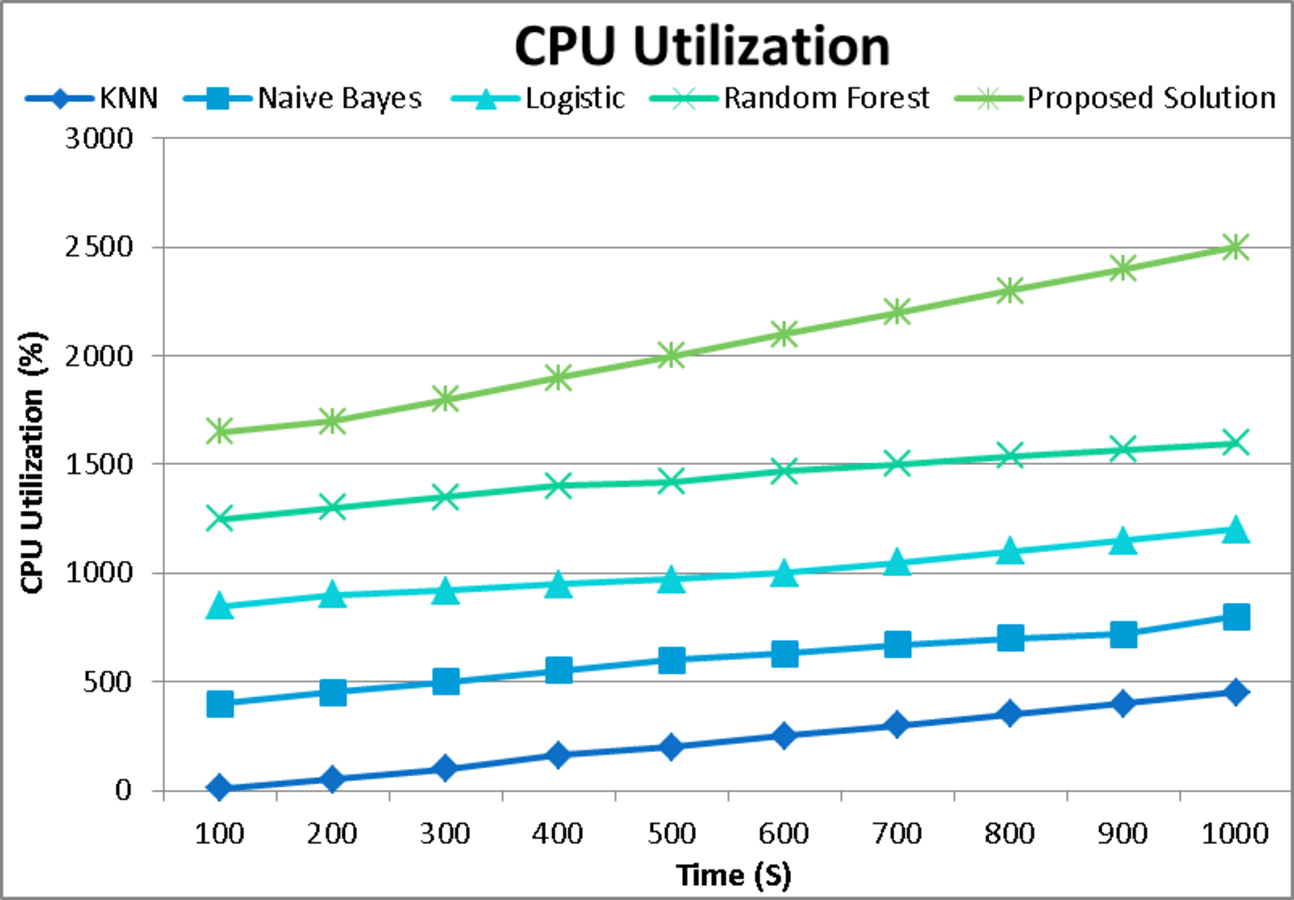

Supplement: Figure S2 [file peerj-cs-10-2257-s003.png]
