# Supplementary material for: Wormhole attack detection and mitigation model for Internet of Things and WSN using machine learning
Source: PeerJ Comput Sci. 2024 Aug 28;10:e2257. doi: 10.7717/peerj-cs.2257 (PMC11419650; doi:10.7717/peerj-cs.2257)
Supplement: Table S1 [file peerj-cs-10-2257-s004.docx]

| Reference. # | ML Techniques | Data Fusion | | WSN | | IoT |
| --- | --- | --- | --- | --- | --- | --- |
| [14]  [15]  [16]  [17] | Traditional  Traditional  Traditional  Traditional | 🗶  🗶  ✓  ✓ | 🗶  ✓  🗶  🗶 | | 🗶  🗶  🗶  ✓ | |
| [18]  [3]  [19]  [20-22]  [23]  [24]  [25]  [This Work] | Traditional  Traditional  Traditional  Traditional  Traditional  Traditional  Traditional  Traditional  SVM and DNN. | ✓  🗶  ✓  🗶  ✓  🗶  🗶  ✓ | ✓  ✓  ✓  ✓  ✓  ✓  ✓  ✓ | | 🗶  ✓  🗶  ✓  🗶  🗶  ✓  ✓ | |
