# Supplementary material for: Wormhole attack detection and mitigation model for Internet of Things and WSN using machine learning
Source: PeerJ Comput Sci. 2024 Aug 28;10:e2257. doi: 10.7717/peerj-cs.2257 (PMC11419650; doi:10.7717/peerj-cs.2257)
Supplement: Table S2 [file peerj-cs-10-2257-s005.docx]

| **Constraints** | **Values** |
| --- | --- |
| Area | 800m * 800m |
| No of nodes  Channel type  Simulator used  Simulation time  Packet size  Data transmission rate  IOT devices  Routing protocol  Execution rounds | 400  Wireless  NS3.37  120 seconds  512/1024 bytes  32 kbps  Secure hybrid routing  150 |
