# Supplementary material for: Wormhole attack detection and mitigation model for Internet of Things and WSN using machine learning
Source: PeerJ Comput Sci. 2024 Aug 28;10:e2257. doi: 10.7717/peerj-cs.2257 (PMC11419650; doi:10.7717/peerj-cs.2257)
Supplement: Table S3 [file peerj-cs-10-2257-s006.docx]

| Aspect | Proposed Solution | State-of-the-Art Methods |
| --- | --- | --- |
| Hardware Requirements (Gupta et al., 2022) | Utilizes existing network infrastructure, minimizes reliance on special hardware or guard nodes | Often requires specialized and expensive hardware or dedicated guard nodes |
| Assumptions (Kuo et al., 2023) | Detects and isolates malicious nodes solely based on network node connectivity information | Relies on assumptions such as network division or insertion of forged links |
| Methodology (Ryu and Kim, 2024) | Employs breadth-first search algorithm to analyze changes in shortest path lengths and determine optimal number of source nodes | Relies on various methods, often with limited scalability or efficiency |
| Source Node Selection (Bhatti et al., 2024) | Uses iterative algorithm to dynamically adjust number of source nodes based on network topology and operational parameters | Selection of source nodes may not be optimized or adapted to network dynamics |
| Detection Process (Ravula et al., 2024) | Incorporates fine-grained statistical analysis, traffic profiling, predictive modeling, adaptive route testing, behavior profiling, self-learning mechanisms, decentralized alert propagation, cross-layer collaboration, and continuous improvement | Often lacks comprehensive integration of advanced detection techniques and feedback mechanisms |
| Scalability and Adaptability (Prasanna and Ramesh, 2024) | Designed to scale and adapt to diverse network configurations, ensuring robust performance and continuous improvement | May lack scalability and adaptability, leading to limited effectiveness in dynamic environments |
